# 9c11tCLA modulates 11t18:1 and 9t18:1 induced inflammations differently in human umbilical vein endothelial cells

**DOI:** 10.1038/s41598-018-19729-9

**Published:** 2018-01-24

**Authors:** Jing Li, Sheng-Ben Hu, Yue-Ming He, Cheng-Fei Zhuo, Ruo-Lin Zhou, Fang Chen, Hong-yan Li, Ze-Yuan Deng

**Affiliations:** 1 State Key Lab of Food Science and Technology, Nanchang University, Nanchang, 330047 China; 2 Institute for Advanced Study, Nanchang University, Nanchang, 330031 China

## Abstract

Endothelial inflammation is recognized as the initial stage of a multistep process leading to coronary heart disease (CHD). Recently, the different effects of industrial *trans* fatty acids (elaidic acid, 9t18:1) and ruminant *trans* fatty acids (vaccenic acid, 11t18:1) on CHD have been reported in epidemiological and animal studies, however, the mechanism was not fully studied. Therefore, the objective of this study was to explore the underlying mechanism by which 9t18:1 and 11t18:1 affect human umbilical vein endothelial cells (HUVECs) inflammation. We found that 9c11t-CLA modulated the inflammation of HUVECs induced by 9t18:1 and 11t18:1. Fatty acid composition, pro-inflammatory factors, phosphorylation of MAPKs, and the TLR4 level in HUVECs altered by 11t18:1 induction, collectively suggest that the bio-conversion of 11t18:1 to 9c11tCLA might be the cause why 11t18:1 and 9t18:1 have distinct influences on endothelial injuries. It was concluded that it is biosynthesis of 9c11t CLA from11t18:1, and the modulation of TLR4-MAPK pathway by 9c11t CLA, which at least partially account for the slight effect of 11t18:1 on endothelial inflammation.

## Introduction

Dietary *trans fatty acids* (TFA) have acquired an unsavory reputation for a long time due to its strong correlation with coronary heart disease (CHD). The dietary sources of TFA are partially hydrogenated oil and ruminant products, with major isomers of industrial TFA (I-TFA) to be elaidic acid (9t18:1, EA) and ruminant TFA (R-TFA) vaccenic acid (11t18:1, VA). Epidemiology studies have demonstrated that I-TFA could provoke CHD^1–4^, hence, limiting TFA intake to less than 1% of total energy was recommended by the World Health Organization. On the other hand, it is not clear whether the R-TFA has the same effect as I-TFA on the cardiovascular system

Current evidence on the effect of R-TFA is inconclusive. Most epidemiological studies suggested an inverse or no association between R-TFA intake and CHD across multiple geographical locations^5–8^. However, three human studies reported a trend for a direct association between R-TFA and CHD^9–11^. Similarly, animal studies showed R-TFA intake is positively correlated with CHD. A male Hartley pigs study^12^ reported that a high intake of R-TFA was as harmful as I-TFA in causing CHD. Another study^13^ pointed that ruminant consumption resulted in an increase of many other trans isomers of 18:1 than VA, and many conjugated fatty acids in addition to rumenic acid, which were potential risk factors for CHD. However, other animal studies suggested that R-TFA had a beneficial effect on CHD. Several studies using VA enriched butter in different animal models demonstrated the hypolipidemic properties of VA^14–17^. In other animal studies, synthetic VA was supplemented to diet in CHD and metabolic syndrome models such as JCR: LA-cp rat, and it was found that VA could lower the plasma triglyceride and cholesterol levels, and attenuated atherosclerotic progression and pro-inflammatory state in dyslipidemia rodent models^18–21^. Inconsistencies among the epidemiological and animal studies may be partially due to the different race, gender, age, diet composition and animal models.

Many studies reported that TFA could induce endothelial cell dysfunction and inflammation, which are integral components of the development and progression of CHD^22^. Harvey^23^ reported that *trans* 18:2 could induce pro-inflammatory responses and endothelial cell dysfunction, and EA could effectively be incorporated into the phospholipid component of endothelial cells and induced pro-inflammatory biomarkers such as elevated intercellular adhesion molecule I (*ICAM-1*) and nuclear factor-κB (NF-κB)^22^. Iwata^24^ also reported that EA could increase NF-κB activation and impair insulin-mediated NO production in endothelial cells while VA was not associated with these responses. Our previous studies observed that endothelial cell injuries induced by VA were significantly weaker than that by EA^25^. These studies indicated that EA could lead to endothelial cell dysfunction, whereas VA had no or weak association with endothelial cell dysfunction. Moreover, VA was reported to be bio-converted to 9c11t-CLA by stearoyl-CoA desaturase (SCD-1, Δ9 desaturase) in human and ruminant. Several animal studies had reported the beneficial effects of CLA intake on atherosclerotic lesions and CVD^26–28^. However, the underlying mechanism by which caused the different effects of VA and EA on cell dysfunction remains unclear.

Therefore, the aim of this study was to explore the occurrence of bio-conversion from VA to 9c11t-CLA, and to evaluate the influence of this bio-conversion on cell dysfunctions mediated by VA and EA.

## Results

### Effect of leptin on the inhibition of Δ9 desaturase (SCD-1)

**Figure 1:**
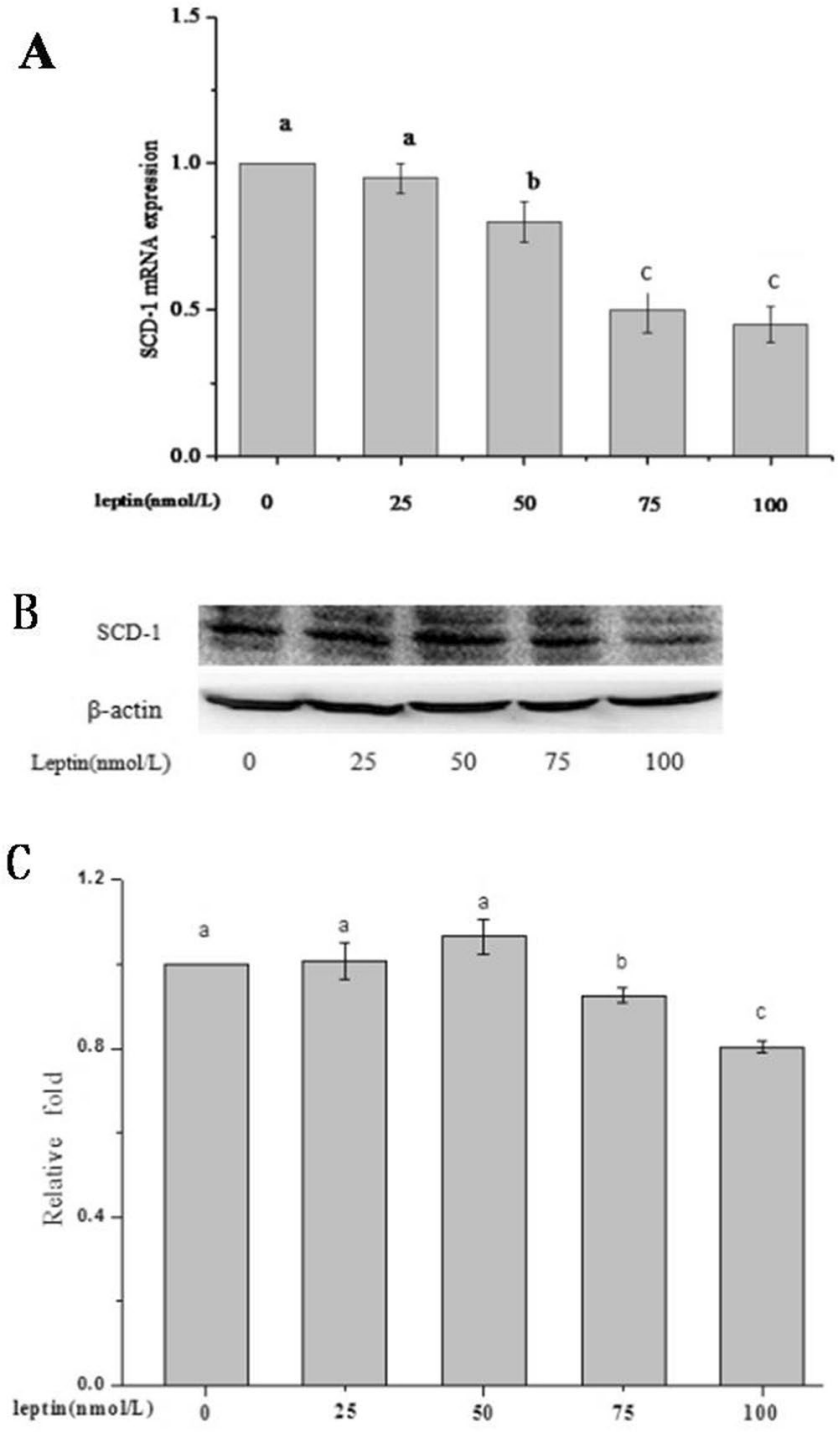
Different concentrations of leptin on the inhibition of Δ9 desaturase (SCD-1). Values labeled with different letters in each set indicate significant differences (*p* < 0.05).

Leptin was reported to down-regulate the mRNA and protein expression of SCD-1, hence, leptin was used to inhibit the bio-conversion of 11t 18:1 into 9c11t CLA in this study. Compared with the control group, the mRNA and protein expression of SCD-1 was reduced when HUVECs was treated with leptin (Fig. 1). When leptin concentration reached 75 nmol/L, the mRNA and protein expressions of SCD-1 in HUVECs were reduced significantly compared with the control group (*P* < 0.05). Hence, 75 nmol/L leptin was chosen as the level to inhibit bio-conversion from 11t 18:1 to 9c11t CLA.

### Effect of 9t18:1 and 11t18:1 on cytotoxicity of HUVECs

**Figure 2:**
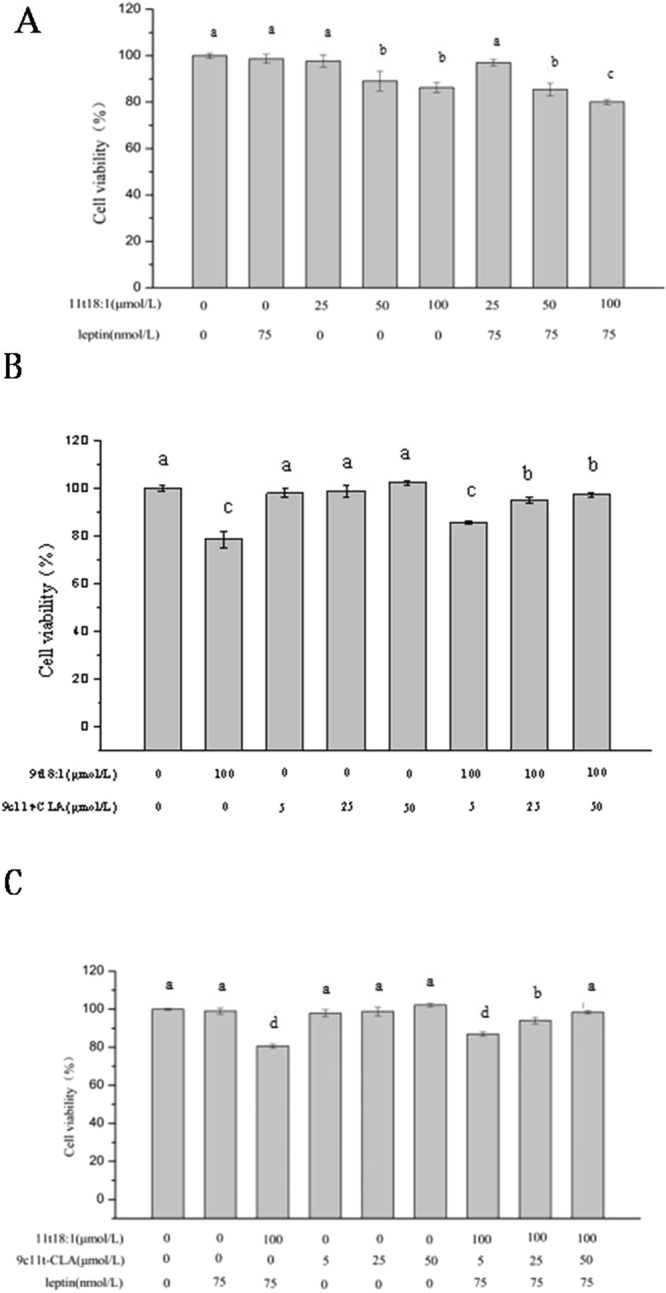
Effect of 9t18:1 and 11t18:1 on cytotoxicity of leptin-treated or non-treated HUVECs. (**A**) The effect of 9t18:1 and 11t18:1 on viability of leptin-treated or non-treated HUVECs. HUVECs were pretreated with or without leptin (75 nmol/L) for 24 h and cultured with 11t18:1(25, 50, 100 μmol/L) for 24 h. (**B**) The effect of 9t18:1on viability of 9c11t-CLA-treated or non-treated HUVECs. HUVECs were pretreated with or without 9c11tCLA (5, 25, 50 μmol/L) for 24 h and cultured with 9t18:1 (100 μmol/L) for 24 h. (**C**) The effect of 11t18:1 + leptin on viability of 9c11t-CLA -treated or non-treated HUVECs. HUVECs were treated or non-treated with 9c11tCLA (5, 25, 50 μmol/L) and then cultured with the group of 11t18:1 (100 μmol/L) + leptin (75 nmol/L) for 24 h. Data were expressed as the mean of three individual experiments ± SD. Values labeled with different letters in each set indicate significant differences (*p* < 0.05).

HUVECs cytotoxicity was determined via measuring the reduction of cell viability by MTT. As shown in Fig. 2A, cell viability showed no significant changes between the control and leptin (75 nmol/L) group within 24 h. After treatment with 11t18:1 and 11t18:1 + leptin for 24 h, cell viability decreased with increased 11t18:1 concentration. However, the cell viability of the 11t18:1 group was significantly greater than that of the 11t18:1 + leptin group (*P* < 0.05) when concentration of 11t18:1 reached to 100 μmol/L.

As shown in Fig. 2B, cell viability showed no difference after treatment with 9c11t-CLA from 5 μmol/L to 50 μmol/L compared with the control group, but cell viability was reduced significantly (*P* < 0.05) in the 9t18:1 group (100 μmol/L). After treatment with 9c11t-CLA + 9t18:1 for 24 h, cell viability showed a positive correlation with 9c11t-CLA concentration, which was significantly greater than the 9t18:1 (100 μmol/L) group at the concentration of 25–50 mol/L 9c11t-CLA (*P* < 0.05).

In Fig. 2C, compared with the control group, cell viability decreased significantly when treated with 11t18:1 (100 μmol/L) + leptin (75 nmol/L) for 24 h (*P* < 0.05). However, compared with the 11t18:1 (100 μmol/L) + leptin (75 nmol/L) group, the cell viability was regained when treated by 11t18:1 (100 μmol/L) + leptin (75 nmol/L) + 9c11t-CLA for 24 h, and the recovery was up to 86.9 ± 1.1% and 98.4 ± 0.9% of the control when the concentrations of 9c11t-CLA were 25 μmol/L and 50 μmol/L, respectively (*P* < 0.05).

### The bio-conversion rate of 11t18:1 into 9c11t-CLA in HUVECs

In HUVECs treated with TFA, the levels of saturated fatty acid (SFA), monounsaturated fatty acid (MUFA) and polyunsaturated fatty acid (PUFA) were decreased, while the total TFA content were significantly increased (*P* < 0.05) (Table 1). In HUVECs treated with 9t18:1, the levels of 9t18:1 significantly increased from 80.48 mg/g to 270.88 mg/g (*P* < 0.05), with no obvious changes in 9c11t-CLA concentration. In HUVECs treated with 11t18:1, the levels of 11t18:1 significantly increased from 73.36 mg/g to 241.52 mg/g (*P* < 0.05), with a significant increase of 9c11t-CLA from 26.16 mg/g to 79.52 mg/g (*P* < 0.05).

**Table 1:** Fatty Acid Composition of HUVECs treated by 9t18:1 and 11t18:1(mg/g total fat).

Fatty acids in lipid fractions	control	leptin	11t18:1(100 μmol/L) + Leptin (75 nmol/L)	11t18:1/(μmol/L)			9t18:1/(μmol/L)		
				25	50	100	25	50	100
SFA	289.44 ± 3.28^ab^	49.68 ± 0.96^a^	200.56 ± 2.72^c^	280.08 ± 4.16^ab^	268.48 ± 6.40^b^	205.44 ± 1.76^c^	290.00 ± 14.24^a^	246.72 ± 16.08^b^	173.60 ± 10.08^d^
ΣcisMUFA	406.32 ± 3.12^a^	416.08 ± 1.44^a^	302.96 ± 2.40^c^	351.44 ± 2.72^b^	305.68 ± 8.80^c^	292.72 ± 3.36^d^	360.56 ± 6.56^b^	349.76 ± 9.68^b^	339.76 ± 14.08^b^
ΣPUFA	91.28 ± 2.88^a^	86.48 ± 0.56^a^	29.20 ± 0.96^c^	72.56 ± 2.48^ab^	64.88 ± 11.84^b^	62.64 ± 0.96^b^	61.36 ± 7.36^b^	56.72 ± 4.48^b^	47.20 ± 4.16^bc^
ΣTFA	12.96 ± 0.16^e^	7.76 ± 0.80^e^	267.28 ± 1.60^a^	95.92 ± 2.40^d^	160.96 ± 1.36^c^	239.20 ± 0.48^b^	88.08 ± 3.04^d^	146.80 ± 3.68^c^	239.44 ± 11.28^b^
ΣCLA	1.36 ± 0.08^d^	0.96 ± 0.40^d^	39.52 ± 1.04^bc^	26.16 ± 2.08^c^	40.48 ± 0.24^b^	79.52 ± 1.04^a^	1.68 ± 0.48^d^	1.12 ± 0.72^d^	2.64 ± 1.20^d^
9t18:1	3.68 ± 0.80^d^	4.00 ± 0.16^d^	3.92 ± 0.08^d^	3.76 ± 0.80^d^	3.92 ± 0.80 ^d^	2.40 ± 1.04^d^	80.48 ± 7.04^c^	137.68 ± 12.24^b^	270.88 ± 9.68^a^
11t18:1	1.92 ± 0.08^e^	2.88 ± 0.80^e^	277.12 ± 0.32^a^	73.36 ± 5.68^d^	120.16 ± 2.48^c^	241.52 ± 0.24^b^	1.52 ± 0.80^e^	0.64 ± 0.16^e^	1.04 ± 0.56^e^
9c11t-CLA	0.56 ± 0.00^d^	ND	36.32 ± 0.80^b^	23.28 ± 2.16^c^	35.44 ± 0.80^b^	70.08 ± 1.84^a^	0.56 ± 0.80^d^	1.12 ± 0.64^d^	0.56 ± 0.96^d^

aMean values ± SD (n = 3). Values in the same row with different letters for each species show significant differences (*p* < 0.05). ΣSFA, total saturated fatty acids, Σcis MUFA, total cis monounsaturated fatty acids, ΣTFA, total trans fatty acids, ΣPUFA, total polyunsaturated fatty acids, ΣCLA, total conjugated linoleic acid.

Based on the levels of 11t18:1 and 9c11t-CLA, the bio-conversion rate of 11t18:1 into 9c11t-CLA was calculated as 23.10 ± 0.79%. Compared with 11t18:1 (100 μmol/L), the 11t18:1 + leptin groups showed a higher level of 11t18:1 but a lower level of 9c11t-CLA (*P* < 0.05). The inhibition rate of bio-conversion of 11t18:1 into 9c11t-CLA by leptin was 48.20 ± 0.78%.

### mRNA expression of *ICAM-I*, vascular cell adhesion molecule I (*VCAM-I*) and interleukin 6 (*IL-6*) in HUVECs treated with 9t18:1 or 11t18:1

**Figure 3:**
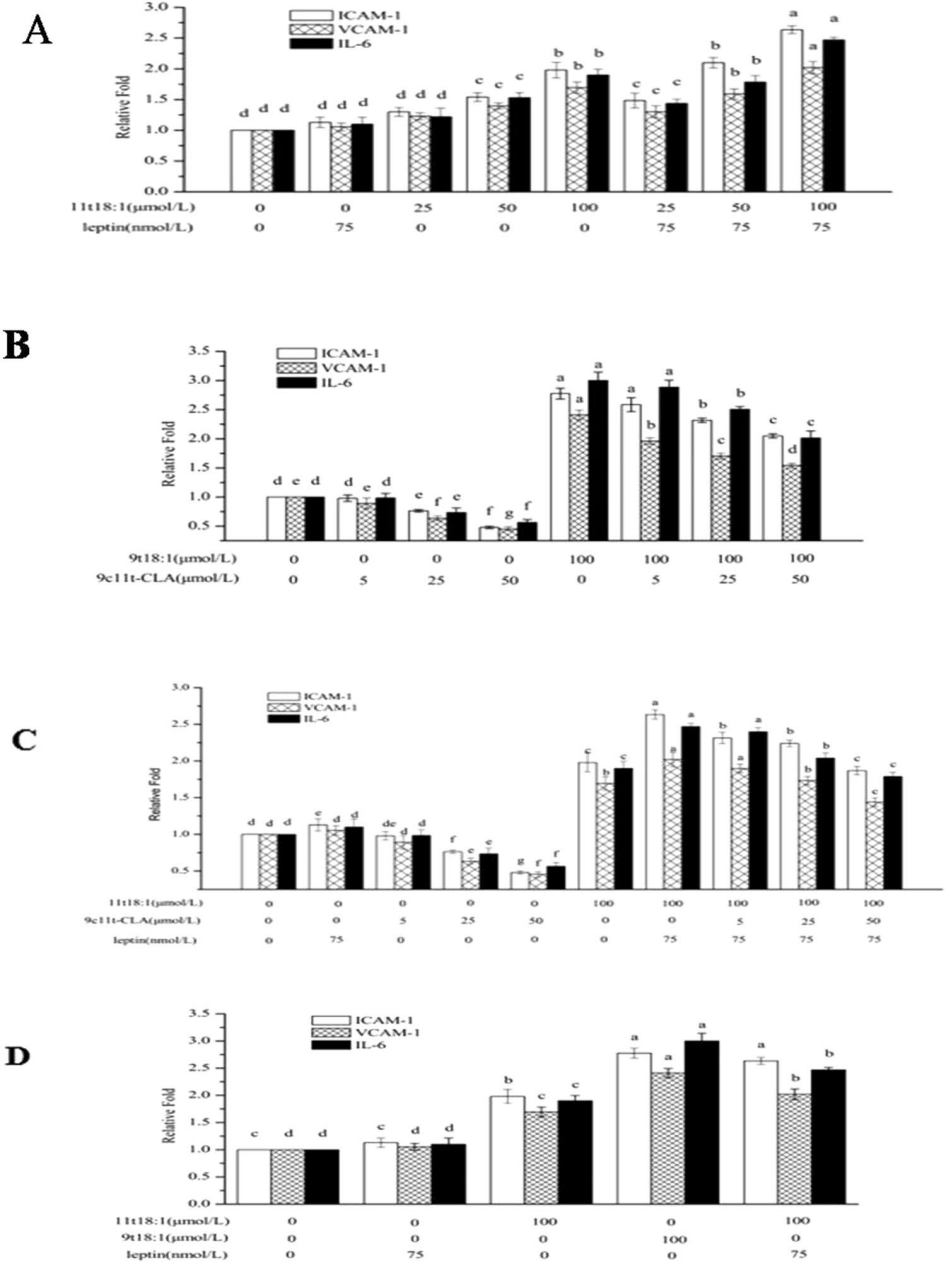
Effect of 9t18:1 and 11t18:1 on gene expression of *ICAM-1, VCAM-1 and IL-6* of leptin/9c11t-CLA treated HUVECs. (**A**) The effect of 11t18:1 on expression of *ICAM-1, VCAM-1 and IL-6* in leptin treated HUVECs. HUVECs were treated or without leptin (75 nmol/L) for 24 h and then cultured with 11t18:1(25, 50, 100 μmol/L) for 24 h. (**B**) The effect of 9t18:1 on expression of *ICAM-1, VCAM-1 and IL-6* in 9c11tCLA treated HUVECs. HUVECs were treated or non-treated with 9c11tCLA (5, 25, 50 μmol/L) and then cultured with 9t18:1 (100 μmol/L) for 24 h. (**C**) The effect of 11t18:1 + leptin on expression of *ICAM-1, VCAM-1 and IL-6* in 9c11tCLA treated HUVECs. HUVECs were treated or non-treated with 9c11tCLA (5, 25, 50 μmol/L) and then cultured with the group of 11t18:1 (100 μmol/L) + leptin (75 nmol/L) for 24 h. (**D**) The effect of 9t18:1 on expression of *ICAM-1, VCAM-1 and IL-6* of leptin treated HUVECs. HUVECs were treated or non-treated with leptin (75 nmol/L) and then cultured with 9t18:1 (100 μmol/L) and 11t18:1 (100 μmol/L) for 24 h. ^a–g^ Data were presented as mean ± SD, values not sharing a common superscript denote significant difference (*P* < 0.05).

The mRNA levels of *ICAM-1, VCAM-1 and IL-6* in HUVECs were analyzed using quantitative real-time PCR. As shown in Fig. 3A, the mRNA expression of *ICAM-1, VCAM-1 and IL-6* of HUVECs in 11t18:1 group and 11t18:1 + leptin group showed a positive relation with 11t18:1 concentration. The mRNA expression of *ICAM-1, VCAM-1 and IL-6* in HUVECs increased significantly in 11t18:1 group at the concentration of 50–100 μmol/L compared with the control group (*P* < 0.05). However, 11t18:1 + leptin group showed significantly higher mRNA levels of *ICAM-1, VCAM-1 and IL-6* than those in the 11t18:1 group (*P* < 0.05) at the same 11t18:1 concentration.

The mRNA expression of *ICAM-1, VCAM-1 and IL-6* of HUVECs decreased significantly in the 9c11t-CLA group at 25–50 μmol/L compared with the control group (*P* < 0.05) (Fig. 3B). However, these genes were expressed at significantly higher level in the 9t18:1 + 9c11t-CLA group than those in the 9c11t-CLA group, although significantly lower than those in the 9t18:1 group (*P* < 0.05) at 25–50 μmol/L of 9c11t-CLA. The mRNA expression of all of these genes in the 9c11t-CLA and 9t18:1 + 9c11t-CLA groups also showed a negative correlation with 9c11t-CLA concentration.

The expression levels of *ICAM-1, VCAM-1 and IL-6* of HUVECs in the 9c11t-CLA and 11t18:1 + leptin + 9c11t-CLA groups were negatively correlated with 9c11t-CLA concentration. The expression of the aforementioned genes in the 11t18:1 + leptin + 9c11t-CLA group was significantly higher than those in the 9c11t-CLA group but significantly (*P* < 0.05) lower than those in the 11t18:1 + leptin group at 25–50 μmol/L of 9c11t-CLA (Fig. 3C).

Compared with the control and leptin group, the gene expression of *ICAM-1, VCAM-1 and IL-6* in HUVECs significantly increased in the 11t18:1, 9t18:1 and 11t18:1 + leptin groups (*P* < 0.05), among which the 9t18:1 group showed the highest level of the above followed by the 11t18:1 + leptin and 11t18:1 groups (Fig. 3D).

### Effect of 9t18:1 and 11t18:1 on MAPKs signaling pathway

**Figure 4:**
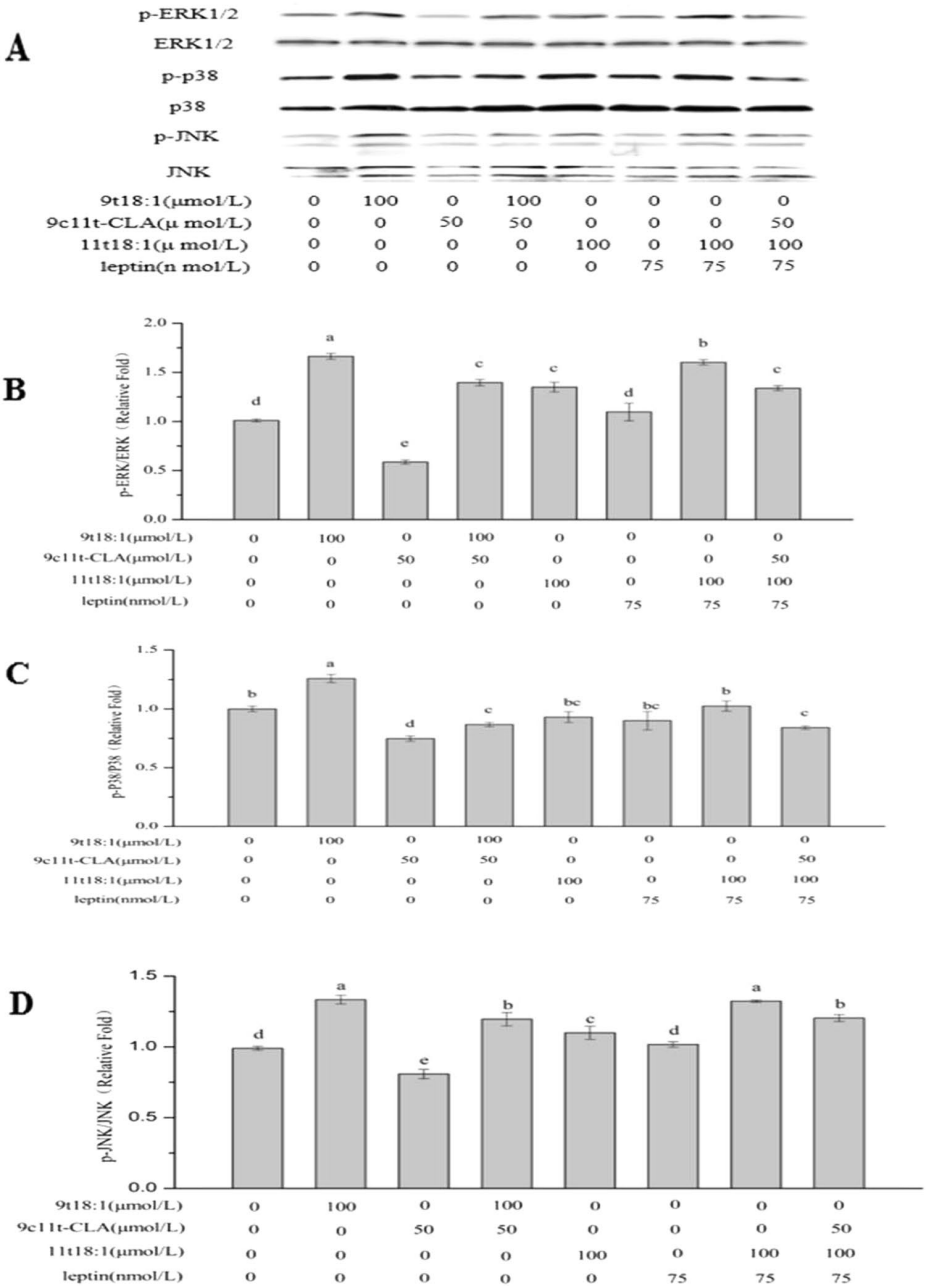
Effect of 9t18:1 and 11t18:1 on MAPKs phosphorylation in HUVECs. HUVECs were treated with 9t18:1 (100 μmol/L), 9c11t-CLA (50 μmol/L), 11t18:1(100 μmol/L), leptin (75 nmol/L), 11t18:1 (100 μmol/L) + leptin (75 nmol/L), 9c11t-CLA(50 μmol/L) + 11t18:1 (100 μmol/L) + leptin (75 nmol/L), or 9c11t-CLA(50 μmol/L) + 9t18:1 (100 μmol/L) for 24 h. (**A**) Electrophoretogram. (**B**) p-ERK Gray analysis diagram. (**C**) p-P38 Gray analysis diagram. (**D**) p-JNK Gray analysis diagram. Values labeled with capital letters indicate extremely significant differences (*p* < 0.01), values labeled with lower case letters indicate significant differences (*p* < 0.05).

The effects of 9t18:1 and 11t18:1 on MAPKs signaling pathway are shown in Fig. 4(A,B,D). The effect of 9t18:1 on the p-ERK/ERK and p-JNK/JNK levels was greater than that of 11t18:1 (*P* < 0.05), but both these two TFA highly significantly up-regulated the p-ERK/ERK and p-JNK/JNK level compared with the control group (*P* < 0.01), whereas in the. 9c11t-CLA group, the levels were highly significantly down-regulated compared with the control group (*P* < 0.01), and the effect in the 11t18:1 + leptin group was significantly lower than in the 11t18:1 group (*P* < 0.05). No obvious change of p-ERK/ERK and p-JNK/JNK were observed between control group and the leptin group. Moreover, obvious down-regulations of p-ERK/ERK and p-JNK/JNK were found in the 11t18:1 + leptin + 9c11t-CLA group compared with the 11t18:1 + leptin and 11t18:1 groups (*P* < 0.05), although the 9t18:1 + 9c11t-CLA group had similar effect as the 9t18:1 group (*P* < 0.05). The level of phosphorylation of p38 (p-p38/p38) in HUVECs is showed in Fig. 4(A,C). The level of p-p38/p38 level down-regulated by 9c11t-CLA (*P* < 0.05), but up-regulated by 9t18:1 (*P* < 0.05) when compared with the control. No significant change of p-p38/p38 was observed among control, 11t18:1, leptin, and 11t18:1 + leptin groups. Moreover, a significant down regulation of p-p38/p38 was found in the 11t18:1 + leptin + 9c11t-CLA group compared with the 11t18:1 + leptin group and the control (*P* < 0.05). Similar trend was found between 9t18:1 + 9c11t-CLA and 9t18:1 (*P* < 0.05). No obvious change of p-p38/p38 level was found between 9t18:1 + 9c11t-CLA- and 11t18:1 + leptin + 9c11t-CLA- treated HUVECs.

### Effect of 9t18:1 and 11t18:1 on TLR4

**Figure 5:**
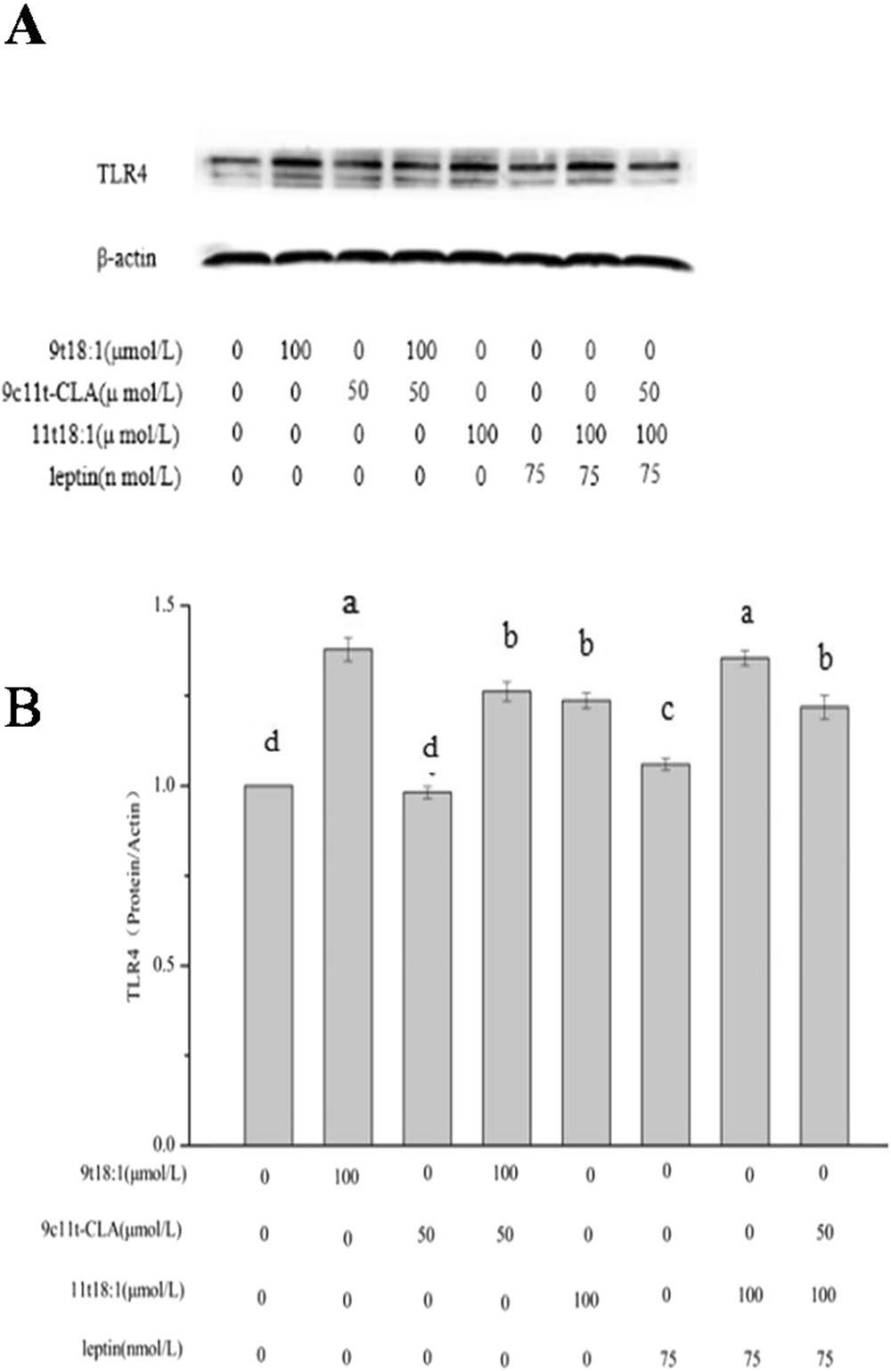
Effect of 9t18:1 and 11t18:1 on TLR4 in HUVECs. HUVECs were treated with 9t18:1 (100 μmol/L) group, 9c11t-CLA (50 μmol/L),11t18:1 (100 μmol/L),11t18:1 (100 μmol/L) + leptin (75 nmol/L), 11t18:1 (100 μmol/L) + leptin (75 nmol/L) + 9c11t-CLA (50 μmol/L) for 24 h. (**A**) Electrophoretogram. (**B**) Gray analysis diagram. Values labeled with different letters in each set indicate significant differences (*p* < 0.05).

Figure 5 (A,B) shows that treatment by 9t18:1 and 11t18:1 significantly up-regulated TLR4 in HUVECs (*P* < 0.05), with a stronger effect seen in 9t18:1 treated cells (*P* < 0.05). TLR4 level was also significantly up-regulated in the 11t18:1 + leptin group compared with the 11t18:1 group or the leptin group. 9t18:1 + 9c11t-CLA had significantly less impact on TLR4 than 9t18:1 (*P* < 0.05) and 11t18:1 + leptin. No significantly change was observed between 9t18:1 + 9c11t-CLA and 11t18:1 + leptin + 9c11t-CLA treated cells.

### Effect of TLR4 inhibitor (TAK242) on the phosphorylation of MAPK by 9t18:1 and 11t18:1

**Figure 6:**
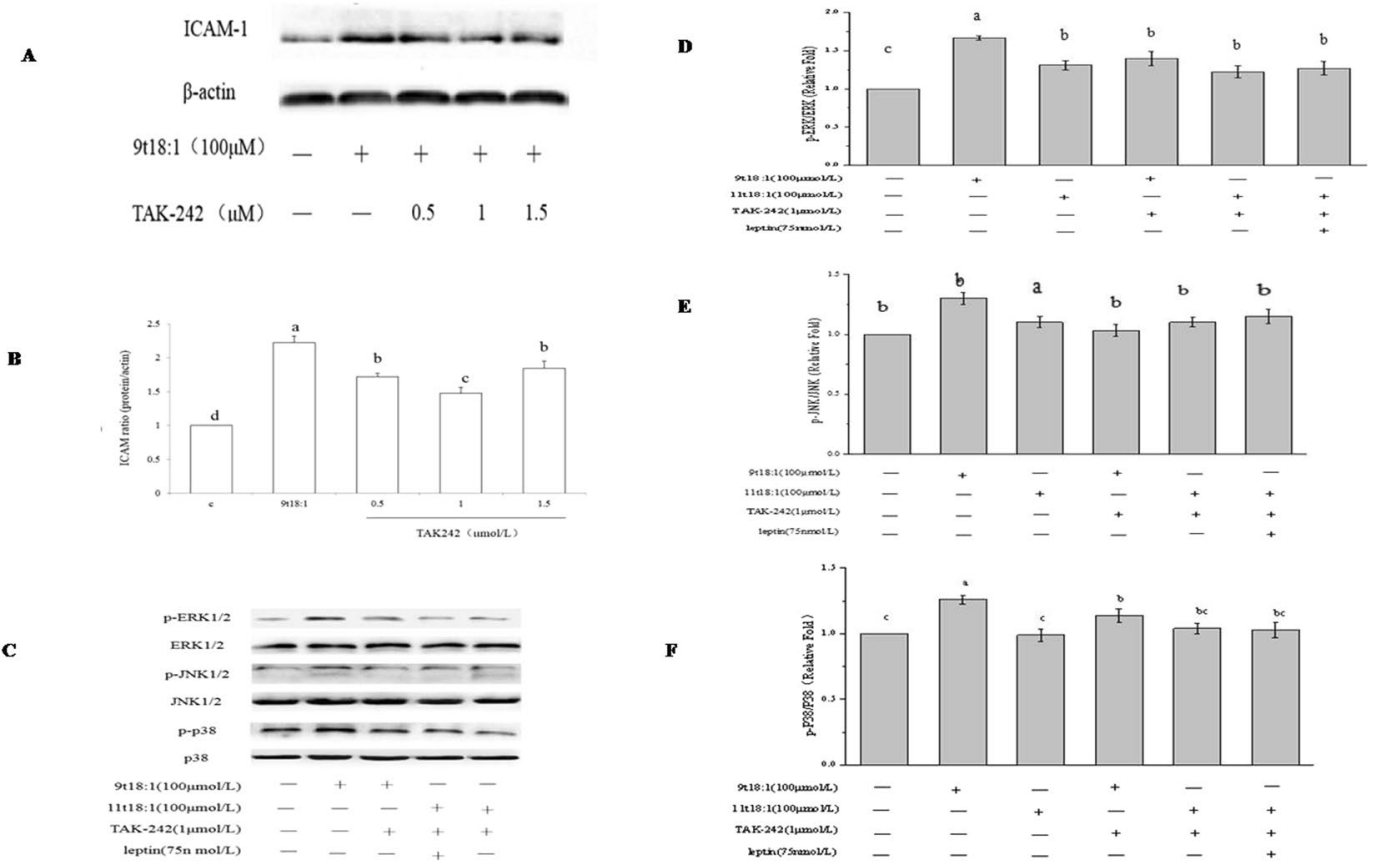
Effect of TAK242 on MAPKs phosphorylation in HUVECs treated with 9t18:1 and 11t18:1. (**A**) Effect of TAK242 on ICAM-1 expression. HUVECs were treated with TAK242 (0.5, 1, 1.5 μmol/L) for 30 min and then cultured with 9t18:1 for 24 h. (**B**) Effect of TAK242 on MAPKs phosphorylation in HUVECs treated with 9t18:1 and 11t18:1. HUVECs were treated with TAK242 (1 μmol/L) for 30 min and then cultured with TFA for 24 h. Values labeled with different letters in each set indicate significant differences (*p* < 0.05).

The expression of ICAM in HUVECs treated with different concentrations (0.5, 1, 1.5 μmol/L) of a TLR4 inhibitor (TAK242) was shown in Fig. 6(A,B). One μmol/L of TAK242 had very significant inhibitory effect on ICAM expression (*P* < 0.05). The p-ERK/ERK, p-JNK/JNK, and p-p38/p38 in the 9t18:1 + TAK242 group were significantly lower than those in the 9t18:1 group (*P* < 0.05), however, no obvious changes were observed in the 11t18:1 + TAK242 and the 11t18:1 + leptin + TAK242 groups when compared with the 11t18:1 group [Fig. 6(C–F)].

## Discussion

Many studies have demonstrated that 9t18:1 can cause negative effect on CHD, whereas 11t18:1 showed no or only weak effect on CHD^7,15,29,30^. Many researchers have attributed the different effects of 9t18:1 and 11t18:1 on health to their different rates of incorporation into membrane lipids^31^, varied degrees of oxidization by liver peroxisomes^32^, various binding extents into the phospholipids in hepatocytes^33^ and to the different effects on cholesterol absorption^34^. In the present study, a remarkable increase of 9t18:1 and 11t18:1 was observed in HUVECs when treated with the two TFA, suggesting they may be preferably absorbed by the HUVECs. Meanwhile, the two *trans* fatty acids may also stimulated the lipids metabolism differently in HUVECs. The levels of SFA, MUFA and PUFA decreased as the concentrations of 9t18:1 and 11t18:1 increased. Particularly, the 9c11t-CLA level was significantly higher in 11t18:1 treated cells than in 9t18:1 treated cells, and the effect by 11t18:1 was dose-response dependent. It was demonstrated that some 11t18:1 could be bio-converted into 9c11t-CLA byΔ9 desaturase in humans and ruminant animals, but 9t18:1 did not have the effect *in vivo*. The bioconversion rate of VA into 9c11t-CLA in HUVECs was 23%, and the conversion rate of 11t18:1 has been reported to be ca. 19% in humans^35^. 9c11t-CLA is one of the main active isomers of CLA, which has been reported to have potent protective functions such as anti-inflammatory, anticarcinogenic, antiadipogenic, antidiabetic, and antihypertensive properties in animal studies^36–39^. Hence, we can reasonably assume that bio-conversion of 11t18:1 to 9c11t-CLA is the main cause of the different influences of 11t18:1 and 9t18:1 on cell dysfunctions.

In order to verify this speculation, leptin was used in our study as an inhibitor of the bio-conversion of 11t18:1 to 9c11t-CLA. Leptin has been reported to inhibit the bio-conversion of 11t18:1 to 9c11t-CLA. In leptin deficient mice, the mRNA expression of *SCD-1*(Δ9 desaturase) was highly up-regulated but normalized after leptin treatment^40^. It was also observed that leptin down-regulated the mRNA expression of *SCD-1*^41^. In HepG2 cells, addition of 70 nmol/L leptin significantly decreased the SCD-1 transcriptional activity and to a greater extent the mRNA and protein level^42^. The first experiment was designed to investigate the effect of VA on HUVECs by pretreating HUVECs with leptin which inhibited the bio-conversion of 11t18:1 to 9c11t-CLA^42^. The second experiment was used to explore the possible modulatory effects of 9c11t-CLA on dysfunctions induced by VA and EA in 9c11t-CLA pretreated HUVECs. HUVECs injury could lead to cell dysfunction and inflammatory response. Many studies demonstrated that intercellular adhesion *ICAM-1, VCAM-1, and IL-6* were typical factors of inflammation and they contributed to the etiology of cardiovascular disease^43,44^. In our study, the mRNA levels of *ICAM-1, VCAM-1, and IL-6* in HUVECs stimulated by VA were significantly up-regulated with leptin treatment, whereas they were remarkably down-regulated by the treatment of 9c11t-CLA. These results point to the anti-inflammatory effects by 9c11t-CLA and support our speculation that the difference in inflammation induced by VA and EA was partially due to the bio-conversion of VA into 9c11t-CLA. Recently, a number of studies have demonstrated a protective effect of VA against the development of CVD. Both VA and 9c11t-CLA have been shown to possess independent beneficial effect on normalizing metabolic abnormalities associated with dyslipidemic and pre-diabetic conditions^7,15,45^. Therefore, increasing VA content in the diet may be a useful approach to maximize the health value of dairy-derived fats in a rat model^45^. The administration of anhydrous milk fat naturally enriched with CLA and VA had beneficial effects on cardiovascular risk biomarkers in spontaneously hypertensive rats^14^. Bassett reported that a vaccenic acid-rich butter protected against atherosclerosis in mice^16^. These studies have confirmed that VA by itself possesses the beneficial effect in animal models, however, the bio-conversion of VA to 9c11t-CLA has been ignored in these studies.

Although both 11t18:1 (even though without adding leptin) and 9t18:1 apparently can cause HUVECs inflammation, the mechanisms of their inflammatory actions are largely unknown. Phosphorylation of MAPKs is known to stimulate the mRNA expression of the inflammatory factors such as *IL-6, VCAM-I and ICAM-I*, and MAPKs of ERK1/2 plays an important role in atherosclerosis. MAPKs of JNK and p38 are closely related to cell apoptosis^46,47^. An up-regulation of MAPKs phosphorylation was found in both EA and VA treated groups, with EA to be a greater modulator than VA. This demonstrated that phosphorylation of MAPKs which contributes to the inflammation of HUVECs was induced by *trans* fatty acids. When bio-conversion of VA to 9c11t-CLA was partially inhibited, phosphorylation of MAPKs was increased. This indicates that VA does not have the same inflammatory effect in HUVECs as EA does. From the results that 9c11t-CLA pre-treated HUVECs significantly decreased phosphorylation of MAPKs to the same level of VA + leptin and EA, and that only 9c11t-CLA pre-treated HUVECs significantly reduced the phosphorylation of MAPKs, it could be concluded that 9c11t-CLA might modulate EA and VA-induced inflammation in HUVECs. Interestingly, the p-ERK/ERK and p-JNK/JNK levels significantly increased in both 9t18:1 and 11t18:1 treated group, compared to the control group (p < 0.01); but p-p38/p38 only significantly increased in 9t18:1 treated group (p < 0.05) compared with the control. These results indicated that in HUVECs, compared to the activation of p-p38/p38, the activation of p-ERK/ERK and p-JNK/JNK by EA or VA is more robust. p-ERK/ERK and p-JNK/JNK can therefore be accounted as the main factors in EA and VA-induced inflammation in HUVECs, as well as better indicators of HUVECs inflammation.

In addition, our study found that TLR4 inhibitor (TAK242) significantly reduced the phosphorylation of MAPKs in HUVECs induced by TFA, suggesting that TFA stimulated phosphorylation of MAPK via TLR4 signaling pathway. Toll-like receptors (TLRs) are pathogen recognition receptors that stimulate inflammatory cells to produce pro-inflammatory factor that orchestrate inflammatory responses. Therefore, pro-inflammatory factors provoked by TLRs activation are a key link in the process of blood vessel damage. TLR4 was the first discovered TLRs in HUVECs. After TLR4 is activated by external stimuli, it triggers a proinflammatory signaling cascade, and stimulates expression of various pro-inflammatory factors^48–50^. Recently, studies have suggested that TLR4 mediated inflammatory responses are associated with the occurrence of atherosclerosis, heart disease, and other vascular diseases^51–53^. Several studies have demonstrated that TLR4 is related to inflammation induced by SFA^54–56^. Our previous study found that TLR4 could be a mediate receptor of inflammation induced by TFA^57^. The present study found that 9c11t-CLA significantly down-regulated EA and VA-induced expression of TLR4, and leptin increased significantly the expression of TLR4 by inhibiting bio-conversion of 11t18:1 to 9c11t-CLA. The results illustrated that 9c11t-CLA could inhibit inflammation induced by EA and VA. Furthermore, we found that VA had much lower inflammatory effect than EA does. One potential reason is that VA can be converted to 9c11t-CLA, which possesses beneficial effect on endothelial cells dysfunction.

In conclusion, bio-conversion of VA to 9c11t-CLA contributes to different inflammatory effects by VA and EA on HUVECs inflammation. The induction of HUVECs inflammation by TFA might be due to the activation of TLR4 in cell membrane, which in turn activates phosphorylation of MAPKs, and the up-regulation of IL-6, ICAM-I and VCAM-I in HUVECs.

## Methods

### Chemical and Reagents

Dulbecco Modified Eagle Medium (DMEM) was obtained from GIBCO (USA). Fetal bovine serum (FBS) was obtained from Biolnd (Aus). The total RNA extraction kit (TRIzol) was purchased from TaKaRa (Jap). Trans Script assay kit was obtained from Beijing Trans Gen Biotech Co. Ltd. (CN) and qPCR assay kit was from Invitrogen (USA). *ICAM-1*, *VCAM-1*, *IL-6* gene primers were obtained from Life Technologies (USA). Anti-ICAM-1, anti-p38, anti-p-p38, anti-SAPK/JNK, anti-p-SAPK/JNK, anti-ERK 1/2, anti-p-ERK1/2, anti-TLR4 and anti-SCD-1 were purchased from Cell Signaling Technology (USA). TAK242 was purchased from Med Chem Express (USA). Recombinant human leptin was purchased from Peprotech (USA). 3-(4, 5-dimethylthiazol-2-yl)-2, 5-diphe-nyltetrazolium (MTT), elaidic acid (9t18:1) and vaccenic acid (11t18:1) were purchased from Sigma (St. Louis, USA) with purity over 99%. The CLA isomer (9-cis, 11-trans-CLA) was purchased from Cayman Chemical Company (AnnArbor, MI, USA) with purity over 96%. 9t18:1, 11t18:1 and 9c11t-CLA were dissolved in 0.1 mM sodium hydroxide solution at 65 °C.

### Experiment design

Experiment one was designed to investigate the effect of VA on HUVECs by inhibiting the bio-conversion of 11t18:1 into 9c11t-CLA with the pretreatment of leptin^15^. HUVECs were performed with addition of one of the following agents: the control group incubated with 0.01 M PBS; leptin group incubated with 75 nmol/L leptin for 24 h; 11t18:1 group incubated with 25, 50, 100 μmol/L 11t18:1 for 24 h, respectively; 9t18:1 group incubated with 100 μmol/L 9t18:1 for 24 h; 11t18:1 + leptin group with incubated with 25, 50, 100 μmol/L 11t18:1 for 24 h, respectively, after treatment with 75 nmol/L leptin for 30 min.

Experiment two was designed to explore the underlying mechanism by which 9c11tCLA improved HUVECs dysfunction induced by VA and EA, respectively. HUVECs were performed with addition of one of the following agents: the control group incubated with 0.01 M PBS, 9c11tCLA group incubated with 5, 25, 50 μmol/L 9c11t-CLA for 24 h, respectively; 9t18:1 group incubated with 100 μmol/L 9t18:1for 24 h; 11t18:1 group incubated with 25, 50, 100 μmol/L 11t18:1 for 24 h, respectively; 9t18:1 + 9c11tCLA group incubated with 5, 25, 50 μmol/L 9c11tCLA for 24 h, respectively, after treatment with 100 μmol/L 9t18:1 for 30 min; 11t18:1 + 9c11tCLA + leptin group incubated with 5, 25, 50 μmol/L 9c11tCLA for 24 h, respectively, after treatment with 100 μmol/L 11t18:1 + 75 nmol/L leptin for 30 min).

Experiment three was designed to explore the influence of TAK242 (TLR4-inhibitor) on MAPK phosphorylation in HUVECs after treatment with EA or VA. HUVECs were performed with addition of one of the following agents: the control group incubated with 0.01 M PBS; 9t18:1 group incubated with 100 μmol/L 9t18:1 for 24 h; 9t18:1 + TAK242 group incubated with 100 μmol/L 9t18:1 for 24 h after treatment with 1μmol/L TAK242 for 30 min; 11t18:1 group incubated with 100 μmol/L 11t18:1 for 24 h; 11t18:1 + TAK242 group incubated with 100 μmol/L 11t18:1 for 24 h after treatment with 1μmol/L TAK242 for 30 min.

### Preparation of HUVECs

HUVECs were maintained in DMEM containing 10% fetal bovine serum at 37 °C in a humidified atmosphere in the presence of 5% CO_2_. Only endothelial cell cultures of less than eight passages and 80–90% confluence were utilized in the present study.

Stock solutions (1 mM) of fatty acids were prepared using fatty acid-free bovine serum albumin^58^. Sub-confluent HUVECs were incubated in DMEM medium supplemented with 10% FBS at 37 °C in a humidified atmosphere of 5% CO_2_ from 24 h to 48 h and then treated with different chemicals according to experiment designs mentioned above. After incubation, the cells were repeatedly washed in PBS (Ca and Mg free).

### Determination of bio-conversion rate of 11t18:1 into 9c11t-CLA

HUVECs were treated with 100 μmol/L of 11t18:1 for 24 h and washed by PBS. The supernatant was removed and the lower sediment tissue was collected into a test tube. Total lipids of cells were extracted using methanol/chloroform (1:3, V/V) and methylated with 2% sodium methoxide. The resultant total fatty acid methyl esters (FAME) was analyzed onto an Agilent Technologies gas chromatograph 6890 equipped with a flame ionization detector and a capillary column of fused silica (100 m × 0.25 mm × 0.20 μm). The temperature was kept at 45 °C for 3 min, increased to 175 °C at a rate of 13 °C /min, kept at 175 °C for 27 min, further increased to 215 °C at a rate of 4 °C /min, and finally kept at 215 °C for 5 min. Injector and detector temperatures were set at 250 °C. Analysis of all peaks was compared with their retention times of FAME standards (GLC-463; Nu-Chek Prep Inc., Elysian, MN, USA). The bio-conversion rate of 11t18:1 into 9c11t-CLA and the inhibition rate of this bio-conversion by leptin were calculated as follow.

1 $$\begin{array}{l}Bio-conversion\,rate=\frac{A}{A+B}\times 100 \% \\ \,A=9c11tCLA(11t18:1group)-9c11tCLA(control)\\ \,B=11t18:1(11t18:1group)-11t18:1(congrol)\end{array}$$

2 $$\begin{array}{c}Inhibition\,rate\,=\\ \,\frac{9c11tCLA(not\,treated\,with\,leptin)-9c11tCLA(treated\,with\,leptin)}{9c11tCLA(not\,treated\,with\,leptin)-9c11tCLA(control)}\times 100 \% \end{array}$$

### Cell viability assay

HUVECs in logarithm period were seeded at a density of 3 × 10^4^–5 × 10^4^ cells/ml into 96-well micro-plates and were cultured at 37 °C under an atmosphere of 5% CO_2_. HUVECs were incubated with different agents according to experiment designs described above. MTT was added to each well after removal of media. After incubation for 4 h, supernatant was removed from each well and cells were dissolved in DMSO. Microplate Reader was used for evaluation of the number of OD changes with the absorbance at 490 nm.

### RNA isolation and quantitative real-time PCR analysis

Total RNA was extracted by Trizol® reagent and converted to complementary DNA (cDNA) with TransScript first-strand cDNA synthesis supermix kit (TransGen) on a thermocycler following the manufacturer’s protocol. QPCR analysis of *scd-1, ICAM-1, VCAM-1, IL-6 and gadph* were performed in an ABI 7900HT QPCR system using Platinum SYBR Green QPCR SuperMix-UDG with ROX. The reaction mixture was subject to the thermal cycling program as follows: heating up to 95 °C in 30 s, followed by 40 cycles at 95 °C for 5 s and 60 °C for 1 min^59^. The expressions of target genes were normalized with that of GAPDH.

### Western blotting analysis

Total cell lysates were extracted by RIPA buffer on ice and the mixture was centrifuged at 20000 g for 15 min at 4 °C. The supernatant containing total protein was collected and stored at −70 °C before use. 80 μg of protein was size-fractionated by 10% SDS-PAGE and transferred to a nitrocellulose membrane. Membranes were blocked in 5% milk at room temperature for 1 h, followed by incubation overnight at 4 °C in the same solution consisting of anti-p38, anti-p-p38, anti-JNK, anti-p-JNK, anti-ERK 1/2, anti-p-ERK1/2, anti-TLR4 and anti-SCD-1 antibodies. Membranes were then washed with TBST followed by incubation at room temperature for another hour with horseradish peroxidase-conjugated IgG antibodies. Membranes were developed with enhanced chemiluminescence (ECL) detection kit and subject to autoradiography on a ChemiDoc XRS instrument^60,61^ (Bio-Rad Laboratory, Hercules, CA).

### Statistical analysis

All experiments were performed in triplicate and values were expressed as mean ± standard deviation (SD). All data were analyzed using one-way analysis of variance (ANOVA) followed by Tukey’s multiple comparison test (Statistical Package for Social Sciences Inc., Chicago, IL, USA). Significance was defined as *p* value less than 0.05.

## Electronic supplementary material

supplementary information
