# Supplementary material for: 9c11tCLA modulates 11t18:1 and 9t18:1 induced inflammations differently in human umbilical vein endothelial cells
Source: Sci Rep. 2018 Jan 24;8:1535. doi: 10.1038/s41598-018-19729-9 (PMC5784167; doi:10.1038/s41598-018-19729-9)

**Supplementary Information**

**9c11tCLA modulates 11t18:1 and 9t18:1 induced inflammations differently in human umbilical vein endothelial cells**

Jing Li^1^, Sheng-Ben Hu^1^, Yue-Ming He^1^, Cheng-Fei Zhuo ^1^, Ruo-Lin Zhou ^1^, Fang Chen^1^, Hong-yan Li ^1^, Ze-Yuan Deng^1,2^*

1. State Key Lab of Food Science and Technology, Nanchang University, Nanchang, 330047, China
2. Institute for Advanced Study, Nanchang University, Nanchang 330031, China

**Corresponding author:**

Professor Ze-Yuan Deng, PhD;

State Key Laboratory of Food Science and Technology, Nanchang University, Nanchang 330047, P. R. China

E-mail: zeyuandeng@hotmail.com

Fax: +86-791-88304402;

Supplementary figure 1

Fig. S1 Full-Length blots

Fig 4A P38


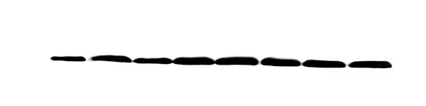


Fig 4A ERK


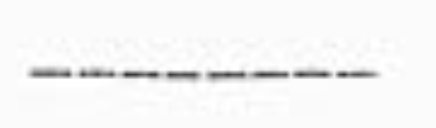


Fig 4A JNK


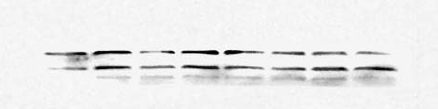


Fig 4A P-ERK


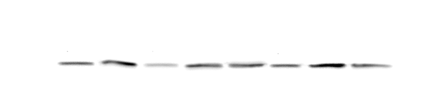


Fig 4A P-JNK


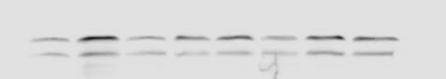


Fig 4A P-P38


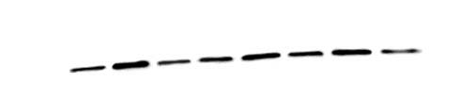


Fig 5A Actin-1


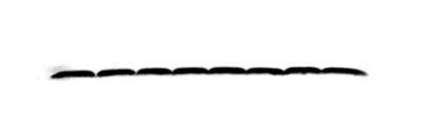


Fig 5A TLR4


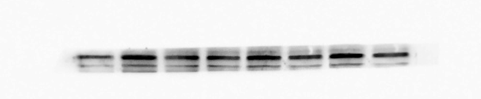


Fig 6A actin -TAK242


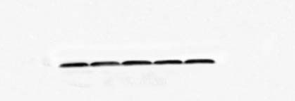


Fig 6A ICAM-TAK242


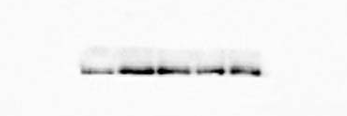


Fig 6C ERK


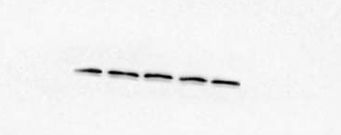


Fig 6C JNK


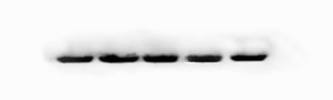


Fig 6C P38


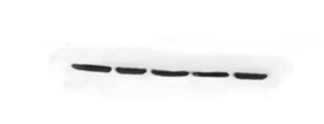


Fig 6C P-ERK


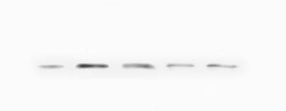


Fig 6C P-JNK


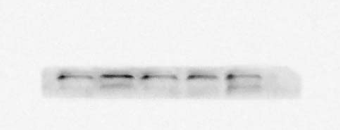


Fig 6C P-P38


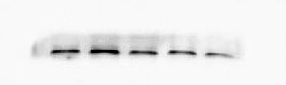

Supplement: Supplementary file 1 — supplementary information [file 41598_2018_19729_MOESM1_ESM.doc]
